# Participation in the state led ‘*Janani Sahayogi Yojana’* public private partnership program to promote facility births in Madhya Pradesh, India: views from private obstetrician partners

**DOI:** 10.1186/s12913-019-4409-2

**Published:** 2019-08-24

**Authors:** Vishal Diwan, Sudhir Chandra Joshi, Kate Jehan, Ayesha De Costa

**Affiliations:** 1 Department of Public Health Sciences, Karolinska Institutet, Stockholm, Sweden; 2 Department of Public Health and Environment, R.D. Gardi Medical College, Ujjain, India; 3 International Centre for Health Research, Ujjain Charitable Trust Hospital and Research Centre, Ujjain, India; 4 Department of Community Medicine, R.D. Gardi Medical College, Ujjain, India; 5 School of Medicine, University of Liverpool, Liverpool, UK

**Keywords:** Public private partnership, C-section, Institutional delivery, Janani Sahayogi Yojana, Madhya Pradesh, India

## Abstract

**Background:**

In Madhya Pradesh, India, the government invited private obstetric hospitals for partnership to provide intrapartum care to poor women, paid for by the state. This statewide program, the Janani Sahayogi Yojana (JShY or maternal support scheme), ran from 2006 to 2012. The partnership was an uneasy one with many private obstetricians choosing to leave the partnership. This paper explores the motives of private obstetricians in the state for participating in the JShY, their experiences within the partnership, their interactions with the state and motives for withdrawal among those who withdrew from the scheme. This study sheds light on the dynamics of a public-private partnership for obstetric care from the perspective of private sector obstetricians.

**Method:**

Fifteen in-depth interviews were conducted with private obstetricians and hospital administrators from eight districts of Madhya Pradesh who had participated in the JShY. A Framework approach was used to analyze the data.

**Results:**

Private obstetricians reported entering the JShY partnership for altruistic reasons but also as way of expanding their practices and reputations. They perceived that although their facilities provided better quality of care than state facilities, participation was risky because beneficiaries were often unbooked and seen as ‘high risk’ cases. The need to arrange for blood transfusions for these high risk women was perceived as particularly difficult. Cumbersome paper work and delays in receiving payments from the state also dissuaded participation. Some participants felt that there was inadequate engagement by the state, and better monitoring and supervision would have helped. The state changed the financial reimbursement arrangements due to a high proportion of Cesarean births in the early years of the partnership, as these were perversely incentivized. This change resulted in a large exodus of private obstetricians from the partnership.

**Conclusion:**

This study highlights the contribution of cumbersome processes, trust deficits and a lack of dialogue between public and private partners. Input from both public and private sectors into the design of a carefully thought through financial reimbursement package for private partners was highlighted as a necessary component for future success of such schemes.

**Electronic supplementary material:**

The online version of this article (10.1186/s12913-019-4409-2) contains supplementary material, which is available to authorized users.

## Background

Attaining universal health coverage (UHC) is an important goal for states aiming to provide fair and equitable health outcomes and improved wellbeing for their populations [1]. Sustainable Development Goal 3 [2] identifies a global target for reaching UHC (Target 3.8), where everyone has access to quality health care without incurring financial difficulty. In India, the National Health Policy [3] advocates public private partnerships (PPPs) as a strategy for improving access to health services for all, and a means to support the global goal of reaching UHC. This is seen as a pragmatic response to India’s vast, rapidly expanding and heterogeneous private health sector. The sector functions largely for profit, and is remunerated by out of pocket payments from patients [4]. By contrast, the public health sector, which has suffered from a degree of underfunding and structural problems, has ceded ground to the private sector in recent decades. The private sector, though poorly regulated, has become the dominant provider of both inpatient (60%) and outpatient health (80%) services across the country [3]. This size and popularity of the private sector, coupled with the limitations of the state sector, have seen PPPs increasingly utilized across a number of areas of healthcare [3, 5, 6].

The Janani Suraksha Yojana (JSY), on which much has been reported [7–9], is the flagship program of the National Rural Health Mission started by the Government of India in 2005 to promote facility births with the ultimate aim of reducing maternal mortality. Under the JSY, women receive a cash transfer on giving birth in a facility. Since the JSY, the proportion of facility births in the country has risen from 26 to 80% between 2005 and 2012 [7]. The JSY program has been implemented largely through public health sector facilities, though a provision exists to accredit private obstetric facilities to implement the program. Thus, the program allows for an element of public private partnership (PPP). The extent of the PPP under the JSY varies in different states of India. Overall, it has accounted for a very small (< 1%) [10] proportion of all the births occurring under the JSY. While there are a number of studies on the JSY in the public sector, the PPP element of the JSY is under-researched [11].

Madhya Pradesh (MP) state has seen one of the sharpest rises in institutional delivery proportions under the JSY [12]. Close to 90% of all facility births have occurred in the public sector; 11% occur in the private sector (22% in urban areas) [13]. The private sector for obstetric services in MP is relatively small and concentrated in the urban areas of the state. It largely operates for-profit; that is, patients pay out of pocket. Care in the private sector is perceived to be superior to care in the public sector, so that even the poor opt for its use in spite of the cost implications [14–16]. Although higher socioeconomic status increases the likelihood of birth in a private health facility [17–19], use of the private sector has increased considerably among lower socioeconomic groups since the 1990s [20].

To further expand access to institutional births in MP, the Department of Health in the state entered a collaborative partnership with the private health sector in 2006, naming the new partnership the Janani Sahyogi Yojana (JShY, or maternal support scheme). Under the JShY partnership, private accredited obstetricians were paid by the state to provide intrapartum care to women below the poverty line. Between 2007 and 2011, 23,000 births occurred in the private sector under the JShY [20]. This paper explores the motives of private obstetricians in MP for participating in the JShY PPP, the benefits and disadvantages of being part of the PPP, their interactions with the state as part of the PPP, and motives for withdrawal among those who withdrew from the scheme. This study explores the dynamics of a PPP for obstetric care from the perspective of private sector obstetricians. The findings of the study will inform the design, feasibility, prerequisites and potential pitfalls for such a partnership for obstetric care in this or other similar low-income settings.

## Methods

### Study setting

MP is one of India’s largest provinces, with a population of 72 million, 72% of which is rural and 31% live below the national poverty line. Facility births in MP stand at 80%, an increase from 26% in 2005 [21]. The state has been the focus of special [22] governmental resources for health under India’s National Rural Health Mission, given that the state has relatively poor socioeconomic and health indicators compared to other Indian states. The infant mortality rate stands at 47 per 1000 live births, the highest in India [23]. Maternal mortality ratio (MMR) is 173 per 100,000 live births [24]. The state is divided into 51 administrative districts, each with a population of 1–2 million [22].

### Obstetric care and the JShY scheme

Obstetric (intrapartum) care in the province is provided by both the public and private sectors, though mostly in the tiered public health sector. The private obstetric sector is confined to the urban parts of the state, largely to district headquarter towns. Though the private obstetric sector was not large, the state government invited a partnership with this sector to further the objectives of the state run JSY program to promote institutional delivery. Under this new *Janani Sahayogi Yojana* (JShY) partnership, the state invited private obstetric facilities meeting certain criteria (mainly the availability of a specialist obstetrician, pediatrician and anesthetist; functional labor room and operating theatre, and capacity of greater than 20 beds) to provide childbirth services to women living below the poverty line at a cost paid by the state. This service was to be free at the point of use.

### Janani Sahayogi Yojana

When the JShY scheme was initiated in 2006, the state announced a fixed reimbursement to private obstetricians of Rs 800 (12 USD) for vaginal births and Rs 4530 (68 USD) for Cesarean births of women living below the poverty line. However, when the proportion of Cesarean sections (C-Section) reported by private obstetricians under the JShY was high – i.e. 41% against a public sector level of 5% [20] – the state suspended the scheme in this form in 2012. The scheme was restarted with a new financial design that did not include a differential reimbursement for vaginal and C-Section births. This study aimed to explore the experiences of obstetricians who had participated in the scheme both before and after the redesign. The scheme is not currently operational in MP.

In the present study, eight districts in Western MP were selected for inclusion in the study. Western MP has a stronger private obstetric sector relative to other parts of the state. The medical teaching hospital which led the present research project was also located in Western MP, therefore facilitating the project in this part of the state was logistically less challenging.

### Participants

In each district, private obstetricians (who are often also hospital managers) and/or hospital managers of private institutions who had participated or currently participate in the JShY program, were identified based on a listing provided of 60 JShY institutions from the state’s Department of Health. In each district, private obstetric facilities were purposively selected to enable the capture of perspectives from different contexts to include: for-profit and charitable hospitals; hospitals of different sizes; facilities that were purely obstetric and those that were part of larger multi-specialty health service providers. Inclusion criteria were that participants should be either obstetricians who ran the facilities, or administrative managers responsible for the JShY PPP at the facility.

### Data collection

Semi-structured interviews (SSIs) were chosen as the most appropriate data collection tool for this study. As our aim was to explore experiences of participation in the JShY, SSIs offer the flexibility needed to prioritize participants’ subjective experiences, whilst providing sufficient structure to cover areas of specific interest to the research question [25]**.** A topic guide developed for this study was prepared and covered participants’ motives for joining the scheme, experience of collaborating under the partnership, benefits and disadvantages from participation in the JShY scheme, and reasons for discontinuation (Additional file 1 - Topic Guide). The obstetricians/managers who agreed to participate in the study were interviewed at their own workplace between August and October 2013. Fifteen interviews were conducted across eight districts. Trained researchers who were members of the RD Gardi Medical College Faculty and had no involvement in the JShY conducted the interviews in either English and/or Hindi. Participant interviews lasted between 45 to 80 min and were audio recorded. Saturation was observed as the interviews progressed so that no more than 15 interviews were deemed necessary.

### Ethics

The study was approved by the institutional ethics committee of R.D. Gardi Medical College, Ujjain, MP, India (permission no. 416, dated 12 September 2014).

### Data analysis

The voice recorded interview sound files were transcribed verbatim by a trained research assistant. The transcripts of Hindi interviews were then translated into English for uniformity. Transcripts were checked for accuracy. The Framework approach [26] to thematic analysis was selected. A draft coding framework based on recurring concepts identified inductively in the transcripts was developed manually. The coding framework was discussed and finalized. Codes were then applied to the remaining transcripts, and framework charts created according to major categories identified within the coding framework. We populated the charts with summarised chunks of data to enable a process of cross-comparison between and within participants’ accounts. The summarized data were discussed by the team, with emergent sub-themes and main themes identified and discussed. Throughout the process, the research team referred back to the raw data and used matrices containing data for each theme to identify similarities and differences across the different participants, districts and institutions.

## Results

We contacted 16 hospitals that currently or previously had participated in the JShY in the study districts. Of these, representatives from 15 hospitals agreed to participate. The hospitals included missionary/charitable hospitals, for profit private hospitals and a teaching hospital. The teaching hospital (and one missionary hospital) were large multispecialty hospitals, the others were smaller, often owned and run by the practicing obstetrician. In eight of these hospitals, the obstetrician-gynecologist working with the program was interviewed while in seven hospitals, the hospital manager/administrator was interviewed. Characteristics of participants are shown in Table 1.

**Table 1:** Characteristics of facilities and participants

Private Facilities	Number	Median bed strength (range)	
Teaching	1	700	
For profit	5	30 (20–100)	
Charitable	3	150 (74–200)	
Participants	Number	Sex	Median Age
Obstetricians	7	2 Male	58
		5 Female	51
Administrators / managers	8	7 Male	45
		1 Female	55

We identified seven main themes to explain private obstetrician and hospital managers’ motives for joining the public-private partnership, the benefits and risks of participation, the contributions they perceived they made and reasons for withdrawing participation.

### Theme I: why join the public private partnership? A mix of altruism and advantage

A common motivation reported by participants for joining the program was altruism. Private providers expressed a desire to provide services to poor women, who could not routinely utilize their services because of financial barriers, as users are often required to pay for services out of pocket.

*“We joined this scheme because there are many poor people who want to come to a private hospital for delivery (childbirth) but they hesitate because of the cost. This program will help poor women to access good facilities.”* **Obstetrician, rural, for-profit hospital**

They also saw themselves as partners in reducing maternal mortality and felt they had a useful role to play.

*“I went for a conference where I heard that Norway has zero maternal mortality […] That’s when I decided to get associated with this scheme to give something back to society.”* **Obstetrician, rural, for-profit hospital**

One mission hospital stated that even though they made a financial loss from participation in the JShY, they continued with participation because of philanthropic missions.

*“There are losses in running this scheme. But we are a mission organization, we join the JShY and similar projects from the government to increase our approach (service) to poor people. But…the hospital has to bear a loss for each patient in the program.”* **Administrator, rural, charitable hospital**

Beyond altruism, providers also perceived advantages for their facilities from participation in the program. These advantages included increasing bed occupancy, gaining experience and building a reputation. One participant perceived the program as being ‘government support’ towards these ends.

*“We thought that our (bed) occupancy would increase, and it increased. Our aim is to decrease delivery (childbirth) costs further and give better care and treatment. When the government gave support to this, it was very good. Patients increased, work increased and people started to know us and our work.”* **Administrator, urban, charitable hospital**

*“We started our hospital in 2006. In a year, we joined the scheme. Due to this, we became very popular and the JShY definitely increased patient inflow and benefitted us.”* **Administrator, urban, for-profit hospital**

### Theme 2: the risks of participation

Participants perceived a risk to participation because of structural issues that stemmed from the absence of necessary life saving measures (such as access to blood) and poor referral practices. They were concerned because of a clustering of high-risk patients at their practices under the program. These were often women whom they had not seen before during pregnancy (‘unbooked’), arrived late and were often in a critical state. In addition, as most potential beneficiaries were poor, they were also likely to be anemic, which necessitated the arrangement of blood for which there was no formal provision under the program.

*“Nearly 50% of pregnant women who come here are highly anemic. We have to arrange for the blood if there is heavy bleeding and we generally give blood to women with less than 7 Hb (hemoglobin level 7 g per deciliter (g/dL) of blood). We had to organize (a) minimum (of) 35 units of blood every month during the scheme. We used to have nearly 100 deliveries per month and nearly 35% cases needed blood.”* **Obstetrician, rural, for-profit hospital**

*“Sometimes we get patients who have no records and they come at 3.00 am morning. They don’t have a sonography report and do not even have a doctor’s prescription. Looking at the plight of the patient, it is impossible to say no and it becomes a problem for us. […] Then (on facing complications in unbooked cases) we start thinking that why are we getting into such problems, and we felt like leaving JShY scheme.”* **Administrator, urban, for-profit hospital**

Another obstetrician described handling cases under the program with requirements for blood that even a public sector facility would find hard to meet.

*“Last Tuesday there was a case - she had a PPH (post-partum hemorrhage) and she landed with hysterectomy. We needed 14 units of blood which is never available in any government hospital”* **Administrator, rural, teaching hospital**

Some private facilities did have small amounts of blood stored at the facility, but this was often inadequate because of the severe anemia with which the women presented.

*“We have to go in for blood transfusion because the patients are referred with very low hemoglobin - 2-3 Hb. We don’t have so much blood available in our blood bank. Also blood components are not available with us.”* **Obstetrician, rural, charitable hospital**

In general, the lack of access to blood for anemic women that private obstetricians reported seeing under the program was strongly emphasized. One private obstetrician had attempted to obtain permissions to have a blood bank at his facility.

*“There is great road block to it (increasing blood bank availability). To get a blood bank license is a big headache. It is lengthy and unnecessarily procedural.*” **Administrator, rural, teaching hospital**

Participants also felt that field health staff at the periphery were not competent enough to be able to refer a patient on time, and so patients were sent in a critical condition. As the participating facilities had functioning operating theaters and full-time gynecologists, they also often received patients who required an intervention for delivery, or had a complication.

*“The ANMs (auxiliary nurse midwives – village-level female health workers employed by the government who are known as the first point of contact between the community and the health services) are not competent enough to diagnose high risk cases. Secondly ANM, Dai (traditional birth attendants) or medical officer or gynecologist in the periphery will hold the patient till the last minute. When finally the patient starts bleeding and on the verge of dying, they are sent here.”* **Administrator, rural, teaching hospital**

*“Generally the patients referred from periphery (remote) places come to us. They are generally referred because they can’t have a normal delivery due to lots of complications. This increases the number of Cesareans. The (regular) patients don’t have much of Cesarean deliveries. In JShY scheme, we had 30–35% Cesareans in which 10–15% cases are always referred from periphery places with lot of complications.”* **Obstetrician, urban, for-profit hospital**

On the whole, private obstetricians who participated in the JShY scheme felt they were taking on a disproportionate degree of risk through their participation. This was attributed to a clustering of ‘unbooked’ (not previously seen) women, who were often anemic or suffered from complications that required expensive and risky surgical intervention.

### Theme 3: the private sector contribution: better quality of care

Despite this perception of risk, the participants strongly asserted the notion that the private sector provided better care to women in need. They rated the technical quality of care as better than that provided in the public sector, as there was consistently specialist (obstetrician) care available and the ability to do a C-Section when needed; these are elements missing from most secondary level public sector facilities. Besides the technical quality of care, the private sector participants argued that they provided a better, cleaner and safer environment for patients.

*“…BPL (below poverty line) families who have little money can come to a private hospital for treatment where the cleanliness, hygiene, care and treatment is better than other government facilities.”* **Obstetrician, urban, for-profit hospital**

An important dimension of quality that was emphasized by the private sector was that patients would not be asked to pay informal fees as rewards, as ‘in the government hospitals’.

*“They (poor people of rural areas) are getting free service and no one in our hospital asks for a reward if a boy baby is born. In government hospital the patient has to give lot of money as rewards to various people. I can say about my department that no one has the guts (dares) to ask money from the patients after the delivery.*” **Obstetrician, rural, teaching hospital**

### Theme 4: cumbersome paperwork and financial procedures deter participation

Private partners spoke of difficulties in routine processes that they had to adopt as a result of participating in the JShY. These included burdensome paperwork, difficulties with obtaining the required documentation from poor patients and delays in obtaining financial reimbursements from the state.

Participating private providers were of the opinion that the amount of administration linked to the program was high and consumed a disproportionate amount of time. In one institution, a person was hired only to handle the paperwork. Providers also took extra care to make copies of relevant documents as their receipt of money from the state was dependent on this. They also mistrusted the state to be careful with the paperwork they had already submitted.

*“There are large number of photocopies to be done of the BPL card, declaration form, birth certificate and the case files. I have to keep one copy here because we get call from government to get details about any patient - they would say that they misplaced the file and they want to recheck the amount. Then I send them my copy to show them the actual cost.”* **Obstetrician, rural, teaching hospital**

There was some frustration among providers who felt that the paperwork load was over-burdensome and had led to considerations of their leaving the partnership.

*“The government is only interested in papers and not in actual treatment. One vitamin tablet has to be registered ten times in the register […] yes, because of this we thought of quitting a lot of time due to this paperwork.”* **Obstetrician, urban, for-profit hospital**

However, there was also an acceptance of the need for some degree of paperwork in the program. Participants acknowledged that this was required to monitor implementation and to prevent misuse.

*“If we need to get some money from the government, we have to follow some process. It is okay, the only thing is they can change some of the things (aspects) in the process for our comfort (convenience). If there is no process, then unwanted people (those who want to take advantage) will come inside (participate in the scheme) and take undue advantages.”* **Obstetrician, urban, for-profit hospital**

Some providers accepted the additional paperwork as a given when it came to working with government, and did not consider leaving the program because if it.

*“No (paperwork did not discourage us from continuing in the program). Generally in all government projects, there is a lot of paper work.”* **Administrator, rural, charitable hospital**

Besides paperwork, private providers also spoke about delays in receiving payments from the state that was owed them under the program. While some hospitals with prior experience of participating in government programs had factored in delays in payments, in other hospitals, it did cause a degree of financial hardship.

*“It was like how it is in every government transaction. It was delayed all the time and was full of obstacles.”* **Obstetrician, urban, for-profit hospital**

*“We have run many government programs and in all the projects money always comes late. So, we are used to it.”* **Administrator, rural, charitable hospital**

*“When everything runs smoothly, there is no problem. But when the government starts delaying the release of funds for 6 months then it becomes a problem. We have to pay salaries and maintain the hospital. Even we have financial crunches. Then it becomes really difficult to manage.”* **Administrator, rural, charitable hospital**

### Theme 5: the problem of a high proportion of C-sections

When the program first began, the state reimbursed private providers differently for vaginal and C-Section births (the latter reimbursed at five times the cost of the former). When the government saw that the proportion of C-Section births seemed disproportionately high under the JShY, the government decided to move to another reimbursement structure. As the state believed that the high C-Section birth rate could be driven by the structure of the financial incentives, the state subsequently changed the incentive structure to a fixed reimbursement per 100 deliveries, regardless of the number of C-Sections and vaginal deliveries. This saw a number of private obstetricians leave the partnership. The private partners in this study had an awareness of this allegation of ‘over-performing’ of C-Sections leveled against them by the government, and justified why they had performed so many.

*“This is a very bad perception (the state’s perception of excessive private sector C-Sections) because Cesarean delivery helps in saving the mother as well as child in many complicated cases. I, being an obstetrician, can tell you that we are doing our job honestly and this is a misperception that we do more unnecessary Cesareans for more money. A few doctors may be doing such malpractices, but majority are doing their work properly and honestly.”* **Obstetrician, urban, for-profit hospital**

*“It is not like that (that we do excessive C-Sections). The cases where we do Cesarean, they are always indicated. In JSY, it is a case of clear cut indicated C-Sections. We can’t trial the patient for labor, when she is already in labor for past 3-4 days. These type of cases come to us at the last minute.”* **Obstetrician, rural, charitable hospital**

Another private obstetrician stated that the nature of the facility being a secondary or tertiary level one meant that there would be higher CS rates which the government was pressuring him to lower.

*“One more problem is that our institution is a secondary or tertiary institution and the number of patients is very high - we have nearly 30–35% Cesarean delivery. They (government) used to pressurize us that we should not have more than 20% deliveries through operation.”* **Obstetrician, urban, for-profit hospital**

A private obstetrician felt the requirements of the program curtailed her ability to decide the appropriate delivery procedure for the patient as an obstetrician.

*“There should not be any stringent rules like this. It depends from patient to patient and conditions differ. I think obstetricians’ observations should be of main importance.”* **Obstetrician, rural, charitable hospital**

Following the new restructuring, a number of private obstetricians left the program, on the grounds that the new payment mechanism was untenable.

*“No, we would have never pulled out from this scheme if it was the earlier terms and conditions.”* **Obstetrician, urban, for-profit hospital**

There also seems to have been some difficulty for providers to understand the rationale of the new reimbursement structure which assumed 15% complicated births and 85% normal births, and paid private obstetricians a package price based on these assumptions.

*“I don’t know why government is paying the same amount for both deliveries […] Cesarean is more expensive than normal delivery which we all know. I don’t think this is a good idea to give equal money for both types (of births).”* **Administrator, rural, charitable hospital**

Others felt that the new reimbursement would not work if they had a high number of C-Sections.

*“The fixed amount of Rs 1, 80,000 ($2,808) for 100 cases was there, which meant Rs 1,800 ($28) for each case. The only problem was if there are Cesarean deliveries, then we would be in a fix.”* **Administrator, urban, for-profit hospital**

Others withdrew because it was not viable given the high number of referred cases they received.

*“They put a condition that in that 100 deliveries, 85 should be normal deliveries and only 15 should be Cesarean delivery. It was not possible to fulfill this condition because we generally have 85% Cesarean delivery and only 15% normal delivery. They never stopped the scheme at our place, but they sent us the new proforma and asked us to comply with it. It is not possible to have maximum (i.e. a majority of) normal deliveries because we generally get referred cases.”* **Administrator, rural, charitable hospital**

### Theme 6: a call for more interaction and supervision from the state

Private sector participants expressed a strong desire for more interaction with the government over the program. This took the form of asking for more supervision, more opportunities for joint interactions and space for clarifications. Some private sector participants believed there should have been much more oversight from the government.

*“If I am the government then I would come for a visit again and again and look into the treatment and the management of the hospital.”* **Obstetrician, rural, charitable hospital**

Many participants referred to the ‘hands off’ approach by the government. They perceived that support from the government was not forthcoming enough to enable the smooth implementation of the program.

*“Government wants you to do the delivery, make a record and send it to them and then they would reimburse the money. The problems which are involved in this full process are overlooked by the government and they never support us with solving these problems.”* **Administrator, urban, charitable hospital**

*“Whom should we call in government? We have many day to day problems and if we go to CMO (Chief Medical Officer) office, they should have time to listen to us and even if they listen to us, they are not going to provide any solution.”* **Administrator, urban, for-profit hospital**

Other participants spoke of the centralization of the program, and that local arms of government close to them were not able to respond to their queries or problems.

*“Regarding delay in funds, we wrote them letters frequently for the fund release. They never wrote to us back but they only verbally told us that even they have not received funds from the head office.”* **Administrator, rural, charitable hospital**

One participant stated that the inability of the local government to make decisions locally led to his institution withdrawing from the program.

*“If the policies were decided at district (local) level, then this scheme would have continued in our place without any conditions.”* **Administrator, rural, charitable hospital**

One participant spoke about the local arm of government trying to help resolve the issues he had, but that they had limited space to maneuver.

*“We never had a problem at local level. The Chief Medical Officer (CMO) was very friendly and all the people in CMO office helped us many times. We used to convey our problems to them and they tried to solve it. They can’t change the schemes which come from the state government, so it was not the fault from the local level.”* **Administrator, urban, for-profit hospital**

Despite the difficulty with the paucity of interactions with the government, there were also some positive experiences.

*“They were very good and they solved many problems. One instance about 2–3 years back was when we had to get nearly INR 1, 100, 000 ($1560) from the CMO and it was getting delayed. We went and presented our case and the money was reimbursed quickly.”* **Obstetrician, urban, for-profit hospital**

### Theme 7: general perception of scheme: a good program overall with caveats

Most participants felt that the partnership was a good one to increase access to institutional delivery among vulnerable groups. Participants felt that the program would help reduce maternal mortality as poor women had access to C-Sections under the program.

Besides the concerns about the clustering of high risk cases among vulnerable women, participants also had concerns about misuse of the program by people who weren’t really below the poverty line but had identification cards to the contrary.

*“Everyone wanted to get benefit of this scheme. All affluent people used to come in cars and they wanted to have the benefit as they had procured a BPL (below poverty line) card through other sources (illegally). The people who were really needy could not get the benefit because they didn’t have a BPL card.”* **Obstetrician, urban, for-profit hospital**

*“They had the cards and we knew that they are from very affluent families, but we had no option and we had to give them the benefit of the scheme.”* **Obstetrician, urban, for-profit hospital**

Because of the above problem, private physicians reported that they were unable to treat patients who were genuinely poor under the program because of an unfair distribution of BPL cards.

*“I have a very poor patient with ruptured uterus and I feel very sorry for her. But I can’t do anything because she doesn’t have a card. I have to submit records so I can’t do anything.”* **Obstetrician, rural, teaching hospital**

Despite these concerns, on the whole private obstetricians felt that the partnership in principle was a good one to promote maternal health.

*“Government’s scheme is very good and it will help in increasing institutional deliveries. For maternal and infant health, this scheme is very necessary.”* **Administrator, urban, for-profit hospital**

## Discussion

This study documents private sector obstetricians’ experiences of participating in a state-led public private partnership in the Indian state of MP. Many private obstetricians situated their decision to join the partnership within an altruistic narrative; at the same time, it was acknowledged as an opportunity to increase bed occupancy in their practices. Participating private institutions saw themselves at risk because of possible clustering of complicated cases, that the poor women they provided care to under the program were often high risk, anemic, in need of blood transfusions, or experienced other complications. There was also a perception that as private sector hospitals, they provided better care than public sector ones in terms of hygiene and overall quality of care. While there was some understanding of the need for detailed paperwork, cumbersome procedures and delayed reimbursements from the state also deterred obstetricians. Some private obstetricians also discussed misuse of the scheme by people who were not considered authentic BPL (below poverty line) card holders, as well as a lack of adequate engagement and supervision by the state in the partnership.

The stated altruistic rationale for joining the JShY was contextualised by participants feeling they had a stake in bringing down maternal deaths or serving disadvantaged clients. Charitable private obstetric facilities stated a mission to work with the underprivileged, and saw their objectives as aligning with those of the state with regard to the JShY i.e. providing access to intrapartum care for poor women. These claims complement the global drive to achieving UHC. For both groups, the declared motivation of altruism was seen as consistent with the ethical code of the Indian Medical Act to which all doctors are required to adhere [27]. As found in studies exploring the Chiranjeevi Yojana program, however, doctors in this study conceded this aspiration needed to be balanced with the imperative to earn a living in a setting where healthcare services in the private sector are delivered as ‘market goods’ [28]. Consistent with findings in studies of the Chiranjeevi Yojana, for-profit private obstetricians also viewed the partnership opportunistically and saw it as a means of building their own practices and gaining experience [28]. Although no private obstetricians mentioned a pecuniary incentive for participation, it is likely that this was an important element, given that a significant number of providers withdrew participation when the financial reimbursement structure was changed. The competing priorities of the obstetricians may ultimately always see the balance tipping in favor of the preservation of the financial health of their businesses. This has implications for an over-reliance on PPPs as a major route to achieving UHC.

Private obstetricians reported a number of difficulties in the partnership which are likely to have contributed to their decision to leave the partnership program. Chief amongst these was the presence of high risk (anemic) women under the program, resulting in the need for blood transfusions as well as a high C-Section rate. The difficulty of accessing blood bank services in India has been documented previously [29]. Given that half of Indian women are anemic [21], that this proportion is likely to be higher in the group of potential JShY beneficiaries, and that postpartum hemorrhage is a major cause of maternal morbidity and mortality in the Indian context [30], it is possible that some elements of such PPPs could work more effectively if there was a sound plan to improving access to blood for women under the program. This could possibly be achieved by creating networks of accredited private hospitals, blood banks in the state and non-governmental sectors.

Public sector C-Section rates have remained steady in MP between 5 and 7% between 2006 and 2016 [31]. Private sector C-Section rates were higher, just before the JShY partnership began at 26% [20]. While the reasons for this are not documented, it is likely that the lack of obstetricians in the public sector and the out of pocket payment system that operates in the private sector contribute to this large difference.

Differential payment for vaginal and C-Section births are widely known to drive C-Sections [32–34]. With its five-fold higher reimbursement rate for C-Sections, the initiation of the JShY program resulted in a sharp increase in C-Section births under the program from 26% in 2007–08 to 41% in 2012, when the state suspended the program [20]. Private obstetricians however justified the high rates of C-Sections on the grounds that their hospitals were specialist hospitals (and offered better quality than state hospitals), and received patients requiring a C-Section that could not receive this service in the public sector. They also suggested a clustering of complications in JShY beneficiaries which would merit the performance of C-Sections. Individual case reviews of JShY beneficiaries’ case reports will need to be performed to objectively assess the necessity of the C-Sections. However, despite these assertions, a number of private obstetricians left the partnership when the incentive for C-Section births was removed, and the program was reintroduced with a fixed block payment per 100 births. Private obstetricians regarded the new fixed block payment as being unviable for their continued participation in the partnership. The fixed block payment per 100 births adopted was similar to the one documented in the Chiranjeevi Yojana program, in which the program designers assumed an 85% uncomplicated vaginal birth rate, 7% C-Section rate and 8% other complication rate [28]. Fixed payments per 100 births were made on this assumption and did not vary depending on the proportion of C-Section births the private obstetrician performed. The change in reimbursement from a differential pro rata payment to a more sophisticated fixed block payment in response to the exceedingly high C-Section rates was a major reason for private obstetricians withdrawing from the scheme.

While some private obstetricians were critical of what they regarded as burdensome paperwork, delays in payment and the inconvenience this caused to some of their practices, there were others who seemed to accept the need to comply with this level of documentation to prevent malpractice. Yet, despite the high levels of paperwork required from the private partners, there was a sense of inadequate engagement by the state in the partnership. Private obstetricians suggested the need for more intense supervision and engagement from the state with regard to the JShY. There was an inability of the local district health administration to make decisions in regard to issues raised by private obstetricians which was also seen as a problem. A lack of dedicated human resources to oversee the implementation of the JShY at the district health office probably resulted in difficulties in resolving implementation challenges experienced by private sector obstetricians in a timely manner. This is consistent with findings from studies exploring similar PPPs, with concern about contact management and scheme monitoring by the state reported in the similar ‘Chiranjeevi Yojana’ PPP [35].

Private obstetricians were also discouraged and expressed irritation at what they observed as misuse of the JShY by disingenuous clients (non-poor clients carrying BPL cards). Providing services free of charge to clients who would otherwise need to pay was a source of discontent for them. For those for whom altruism was a key motivating factor to participate, the misuse of the program by non-poor women possibly diluted their altruistic zeal for participation. In India, BPL cards are issued based on population surveys at regular intervals and remain controversial as 40% of cards are reportedly issued to non poor families [36]. Resolving difficulties associated with the cards’ use and misuse are beyond the scope of the JShY program, or the wider health system. This issue would benefit from examination at the central government level.

### Implications of our findings

There are important implications for this and similar PPPs of the reflections of private obstetricians on this partnership, such as the JShY requiring detailed thought through planning. The rationale for the PPP needs careful consideration, importantly the size and location of the private partners. In MP, the private obstetric sector is small, providing less than a tenth of all facility birth services [13]. Further, it is concentrated in urban areas where there are functional public hospitals. The rationale for what the gains might be from such a partnership in terms of increased access is unclear. Reports indicate a very small contribution of the JShY [20] (< 1%) to the large number of facility births already occurring under the JSY program in MP. Also, the Chiranjeevi Yojana PPP in Gujarat had a significant number (though not a majority) of private obstetric partners outside the large cities [37] where public sector obstetric services were not available. This provided geographic access to skilled care and reduced financial access barriers simultaneously. MP state is different from Gujarat in the size and distribution of its private obstetric sector (private obstetricians tend to be concentrated in the large cities), and this needs consideration while planning a partnership. This also points to the contextual nature of PPPs as a suitable means for achieving UHC, a finding consistent with the UHC literature [38]. A country as vast as India is unlikely to benefit from a one-size-fits-all policy solution; even smaller country contexts are likely to have a heterogeneity of health care contexts which demand careful appraisal in terms of devising suitable policy responses. Whilst PPPs remain a popular strategy in low income contexts for bridging the divide between public and private sectors, in some contexts, strengthening the public sector may be a more appropriate strategy to attaining UHC than adherence to PPPs.

Where it is a suitable choice, the state needs to pay particular attention to designing an appropriate reimbursement scheme and engage in consultations with a broad set of stakeholders, including private partners or their representatives to obtain buy-in and understanding of the rationale for the reimbursement plan. In addition, resources for managing, supervising and coordinating a PPP program from the state need to be carefully considered and factored in. Although the evidence clearly indicates that differential payment for individual cases would lead to an over-performance of C-Sections, the state chose this as the reimbursement method. Policy makers will benefit from discussions with academic, professional bodies, private obstetricians, NGOs and other stakeholders when planning an appropriate reimbursement mechanism.

Extensive paperwork is a poor replacement for close monitoring and supervision. It is important for both parties in the partnership to adequately engage and for the state to provide the leadership required of it. As this was a state led program, effective and timely responses to issues raised by the private partners would have gone a long way to building trust and strengthening the implementation of the program. State led PPP programs in India have often relied on existing health department staff taking on additional work over their routine tasks to oversee these programs, which results in inadequate oversight and engagement. Investment in a PPP program also requires investment in human resources to manage and oversee the program.

Structural issues within the health system and beyond need attention prior to beginning a PPP, although they cannot always be dealt with and redressed entirely, they need attention to avert undermining the partnership program. In the case of the JShY, given its focus on intrapartum care and the magnitude of the maternal morbidity / mortality from anemia and postpartum hemorrhage, access to blood was clearly an important element to secure. The structural issues that compromise access to blood in the health system also adversely affected the partnership program. Similarly, the inappropriate distribution of BPL cards to non-poor households is a problem that would compromise the objectives of the partnership program to provide coverage to those who need it most. This has led to a demoralization of the private partners, some of whom contend that they participate for altruistic reasons. Arguably, if India is to achieve its desired goal of attaining UHC through this (and other) means, then substantial reform may be required across both private and public sectors of healthcare. Success of such schemes requires a strengthened and suitably financed public sector to confidently co-design, deliver and monitor the programs in tandem with their private sector partners, so that it may be of mutual benefit to both partners and the populations whose ultimate aim it is to serve. At the same time, evidence suggests that increasing the reach of health care coverage does not result in better outcomes by default [39, 40]. Expansion may not improve outcomes if quality of care is lacking, effective treatment not available, or the social conditions in which a neglect of poor and marginalized women’s health is permitted to prevail.

### Strengths and limitations

We conducted these interviews across eight districts of MP to reflect views from across the state. We selected facilities to include various types of private obstetric partners under the JShY – teaching hospitals, charitable mission hospitals and for profit private facilities. This variation allowed us to capture common shared experiences around participation in the JShY as well as areas of difference. The research team has had many years of experience researching in the state, the first two authors live and work in MP which allowed them to quickly establish a rapport, generate discussion and gather detailed accounts of participation in the JShY. The last author has lived and worked with maternal health in the project over the last decade.

Although issues raised by private obstetricians that either encourage or deter participation in a PPP program to promote facility births are very context specific, the issues raised in our study in MP demonstrate a number of similarities to those raised by private obstetricians participating in the Chiranjeevi Yojana PPP in Gujarat. This would indicate the transferability of our findings to other PPP programs to promote intrapartum care in other parts of the country.

Despite the strengths of varied views across the state, the study has limitations. The absence of the perspective of the state side of the partnership is a limitation as is the potential for social desirability bias with reference to altruism as a motivating factor for participation.

## Conclusion

Private obstetricians report that altruism and the possibility to build and expand on clinical practices are important drivers for participation in a PPP to raise facility births. However, deterrents to participation included changes in the reimbursement structures by the state, the clustering on high risk women among program beneficiaries, poor engagement by the state, cumbersome procedures and delays in reimbursements and misuse of the scheme by non-poor families. Participatory designing of carefully debated financial reimbursement packages for private partners so that there are no perverse incentives is key. The state needs to ensure adequate human resource commitments to allow appropriate levels of engagement in, monitoring and oversight of the PPP program. This is particularly important so that women receive the care most appropriate to their needs and unnecessary C-sections are avoided. Also broader structural issues like more accurate BPL identification need attention so that programs intended for these groups are not undermined. Our findings provide important considerations for policy makers and planners in the design and implementation of PPP in the area of maternal and child heath in low-middle income settings.

## Additional file

Additional file 1: Topic Guide. (DOCX 21 kb)

## Data Availability

Due to ethical and legal restrictions, all inquiries should be made with The Chairman, Ethics Committee, R.D. Gardi Medical College, Agar Road, Ujjain, India 456006 (Emails: iecrdgmc@yahoo.in, uctharc@bsnl.in), giving all details of the publication. Upon verification of authenticity of the inquiry, the data will be made available. For reference, please quote ethical permission no. 416, dated September 12 2014.

## Abbreviations

BPL: Below poverty line
C-Section: Cesarean sections
JShY: Janani Sahayogi Yojana
JSY: Janani Suraksha Yojana
MP: Madhya Pradesh
PPP: Public private partnership

## References

[1] Kieny MP, Bekedam H, Dovlo D, Fitzgerald J, Habicht J, Harrison G (2017). Strengthening health systems for universal health coverage and sustainable development. Bull World Health Organ.

[2] World Health Organization (2019). SDG 3: ensure healthy lives and promote wellbeing for all at all ages.

[3] Ministry of Health and Family Welfare, Government of India, National Health Policy 2017. http://pib.nic.in/newsite/PrintRelease.aspx?relid=159376. Accessed 23 Mar 2019.

[4] van Doorslaer E, O’Donnell O, Rannan-Eliya RP, Somanathan A, Adhikari SR, Garg CC (2007). Catastrophic payments for health care in Asia. Health Econ.

[5] World Health Organization: India country profile 2014. http://www.who.int/countries/ind/en/. Accessed 24 Mar 2019.

[6] Kasthuri A (2018). Challenges to healthcare in India - the five A’s. Indian J Community Med.

[7] Randive B, Diwan V, De Costa A (2013). India’s conditional cash transfer programme (the JSY) to promote institutional birth: is there an association between institutional birth proportion and maternal mortality?. PLoS One.

[8] Sabde Y, Diwan V, Randive B, Chaturvedi S, Sidney K, Salazar M (2016). The availability of emergency obstetric care in the context of the JSY cash transfer programme in Madhya Pradesh, India. BMC Pregnancy Childbirth.

[9] Sidney K, Ryan K, Diwan V, De Costa A (2014). Utilization of a state run public private emergency transportation service exclusively for childbirth: the Janani (maternal) express program in Madhya Pradesh, India. PLoS One.

[10] United Nations Population Fund. Concurrent assessment of Janani Suraksha Yojana (JSY) in selected states. https://india.unfpa.org/sites/default/files/pub-pdf/JSYConcurrentAssessment.pdf. Accessed 6 June 2019.

[11] Chaturvedi S, Randive B (2011). Public private partnerships for emergency obstetric care: lessons from Maharashtra. Indian J Community Med.

[12] Lim SS, Dandona L, Hoisington JA, James SL, Hogan MC, Gakidou E (2010). India’s Janani Suraksha Yojana, a conditional cash transfer programme to increase births in health facilities: an impact evaluation. Lancet.

[13] Ministry of Home Affairs Government of India, New Delhi (2013). Annual health survey bulletin 2012-2013: Madhya Pradesh.

[14] Nair H, Panda R (2011). Quality of maternal healthcare in India: has the National Rural Health Mission made a difference?. J Glob Health.

[15] Raman S (2014). Faith, trust and the perinatal healthcare maze in urban India. Health Cult Soc.

[16] Skordis-Worrall J, Pace N, Bapat U, Das S, More NS, Joshi W (2011). Maternal and neonatal health expenditure in Mumbai slums (India): a cross sectional study. BMC Public Health.

[17] Bhatia J, Cleland J (1995). Self-reported symptoms of gynecological morbidity and their treatment in south India. Stud Fam Plan.

[18] Kesterton AJ, Cabral de Mello M (2010). Generating demand and community support for sexual and reproductive health services for young people: a review of the literature and programs. Reprod Health.

[19] Thind A, Mohani A, Banerjee K, Hagigi F (2008). Where to deliver? Analysis of choice of delivery location from a national survey in India. BMC Public Health.

[20] Bogg L, Diwan V, Vora KS, DeCosta A (2016). Impact of alternative maternal demand-side financial support programs in India on the caesarean section rates: indications of supplier-induced demand. Matern Child Health J.

[21] Ministry of Health and Family Welfare Government of India (2016). National Family Health Survey 2015–16 (NFHS-4): states factsheets.

[22] Primary Census Abstract: Madhya Pradesh (2011). Government of India.

[23] Registrar General of India (2017). Sample registration system. Government of India. SRS Bulletin. Estimated birth rate, death rate, natural growth rate and infant mortality rate, 2016.

[24] National Institution for Transforming India. Maternal Mortality Ratio (MMR) (Per 100000 Live Births). https://www.niti.gov.in/content/maternal-mortality-ratio-mmr-100000-live-births. Accessed 15 June 2019.

[25] King NHC (2010). Interviews in qualitative research.

[26] Ritchie J, Spencer L, O’Connor W, Ritchie J, Lewis J, Nicholls CM, Ormston R (2003). Carrying out qualitative analysis. Qualitative research practice: a guide for social science students and researchers.

[27] Medical Council of India (2002). Code of ethics regulations.

[28] Ganguly P, Jehan K, de Costa A, Mavalankar D, Smith H (2014). Considerations of private sector obstetricians on participation in the state led “Chiranjeevi Yojana” scheme to promote institutional delivery in Gujarat, India: a qualitative study. BMC Pregnancy Childbirth.

[29] Ramani KV, Mavalankar DV, Govil D (2009). Study of blood-transfusion services in Maharashtra and Gujarat states, India. J Health Popul Nutr.

[30] Registrar General of India, Government of India (2006). Sample registration system. Maternal mortality in India 1997–2003; trends cause and risk factors New Delhi Ministry of Home Affairs.

[31] Gaur A (2016). C-section deliveries rise in private hospital across state Indore.

[32] Bertollini R, DiLallo D, Spadea T, Perucci C (1992). Cesarean section rates in Italy by hospital payment mode: an analysis based on birth certificates. Am J Public Health.

[33] Grant D (2005). Explaining source of payment differences in U.S. cesarean rates: why do privately insured mothers receive more cesareans than mothers who are not privately insured?. Health Care Manag Sci.

[34] Huang K, Tao F, Bogg L, Tang S (2012). Impact of alternative reimbursement strategies in the new cooperative medical scheme on caesarean delivery rates: a mixed-method study in rural China. BMC Health Serv Res.

[35] Bhat R, Singh A, Maheshwari SK, Somen S. Maternal health financing- issues and options: a study of Chiranjeevi Yojana in Gujarat. Ahmedabad: Indian Institute of Management; 2006. Contract no.: working paper number: 2006-08-03.

[36] Ram FRU. Understanding the distribution of BPL cards: all-India and selected states. Econ Polit Wkly. 2009;xliv(7):66–71.

[37] Iyer V, Sidney K, Mehta R, Mavalankar D (2016). Availability and provision of emergency obstetric care under a public-private partnership in three districts of Gujarat, India: lessons for universal health coverage. BMJ Glob Health.

[38] Shroff ZC, Rao KD, Bennett S, Paina L, Ingabire MG, Ghaffar A (2018). Moving towards universal health coverage: engaging non-state providers. Int J Equity Health.

[39] Kruk ME, Gage AD, Joseph NT, Danaei G, Garcia-Saiso S, Salomon JA (2018). Mortality due to low-quality health systems in the universal health coverage era: a systematic analysis of amenable deaths in 137 countries. Lancet.

[40] Odendaal WA, Ward K, Uneke J, Uro-Chukwu H, Chitama D, Balakrishna Y (2018). Contracting out to improve the use of clinical health services and health outcomes in low- and middle-income countries. Cochrane Database Syst Rev.

