# Supplementary material for: Participation in the state led ‘Janani Sahayogi Yojana’ public private partnership program to promote facility births in Madhya Pradesh, India: views from private obstetrician partners
Source: BMC Health Serv Res. 2019 Aug 24;19:599. doi: 10.1186/s12913-019-4409-2 (PMC6708218; doi:10.1186/s12913-019-4409-2)
Supplement: Supplementary file 1 — Topic Guide. (DOCX 21 kb) [file 12913_2019_4409_MOESM1_ESM.docx]

**Additional file 1: Topic Guide**

**Study:** Participation in the state led ‘*Janani Sahayogi Yojana’* public private partnership program to promote facility births in Madhya Pradesh, India: views from private obstetrician partners

**Ice breaker**

- When did you/your institution join JShY?
- How long did your participation continue?

**1: Joining the scheme**

Why did you choose to join the scheme?

What were the advantages for your hospital in doing so? How did you join the scheme?

*Prompts:*

- Who invited you to join the scheme?
- What role did the government play?
- Who invited you? Who encouraged you?
- What were the considerations that passed through your mind before you committed yourself to partnering?

**2: Experience of the program**

Can you tell me about your experience of participating in the program?

*Prompts:*

- What did participating do for the obstetric patient turnover in your hospital?

(Any changes? By how much? Why/why not?)

- What was your experience with the administrative processes involved in participating in JShY (e.g. paperwork, receipt of funds, payment to mothers, how the money flow worked)
- What was your experience regarding any clinical changes (e.g. C-sections)?
- Any challenges that emerged during the implementation of the scheme?
- How were these approached? (e.g. How was attention paid to these? What was the outcome?)
- What was the responsiveness of government staff to the problems you had, while implementing the scheme?

**3: Personal perceptions**

Can you tell me about your own personal perspective regarding private sector involvement in the JShY scheme?

- What were the benefits of the scheme for your institute, in your view? Disadvantages?
- How did the private sector as a whole benefit from participating in JSY? Not benefit? How/why?

**4: End of the program**

The private partnership in JSY was ultimately stopped by the government.

- If the scheme had continued, what would you have done? (Remained with the scheme? Withdrawn anyway? Why? Why not?
- Why do you think the government made a decision to stop the partnership? What did you think of this decision?
- If the scheme were to run again, what would you change? Keep the same?
